# Technology-Related Trauma in Sexual and Reproductive Health Digital Technologies: Grounded Theory Study

**DOI:** 10.2196/79365

**Published:** 2025-11-19

**Authors:** Abdul-Fatawu Abdulai, Janell Cosco Josephs, Adrian Guta, Mark Gilbert, Vicky Bungay

**Affiliations:** 1 School of Nursing, University of British Columbia, T201-2211 Wesbrook Mall, Vancouver, BC, V6T 2B5, Canada, 1 6048227214; 2 School of Social Work, University of Windsor, Windsor, ON, Canada; 3 British Columbia Centre for Disease Control, Vancouver, BC, Canada

**Keywords:** emotional trauma, technology-related trauma, sexual and reproductive health, digital health technologies, grounded theory

## Abstract

**Background:**

Digital health technologies are increasingly used as complementary and alternative means of seeking sexual and reproductive health services. These platforms now play a critical role in facilitating services such as contraception counseling, abortion care, sexually transmitted infection testing and treatment, and fertility-related support, particularly for individuals who face barriers to in-person care. Despite their increasing prevalence, there is an emerging concern that such platforms could inadvertently trigger or perpetuate trauma among end-user patients. This risk is particularly salient for individuals from equity-deserving populations who already navigate stigma, discrimination, or prior traumatic experiences in health care settings.

**Objective:**

This study aimed to develop a theoretical account of how digital health technologies can cause or perpetuate emotional trauma among people who seek technology-based sexual and reproductive health services.

**Methods:**

We used the Charmaz constructivist grounded theory approach by conducting interviews with 25 participants who have used government and other regulated digital health platforms (ie, web-based platforms and mHealth apps) to access sexual and reproductive health information or services including sexually transmitted infection testing, contraception, and abortion. Data analysis occurred alongside data collection, and data were analyzed inductively using open, axial, and theoretical coding.

**Results:**

We developed an explanatory model that shows that technology-related harm can occur in two main ways: (1) digital platform design features (ie, navigation challenges, data and security breaches, and inappropriate display of content) and (2) digital platform–related interpersonal interactions (targeted campaigns and depersonalized digital health interactions). While these activities can cause harm to users in general, they are more likely to result in emotional trauma for individuals with prior traumatic experiences and in emotional discomfort for those without such histories.

**Conclusions:**

Web-based platforms provide opportunities for advancing access to sexual and reproductive health and services. At the same time, these technologies can also serve as conduits through which trauma can be triggered, perpetuated, and exacerbated. While technology-related trauma could occur unintentionally via design choices, some activities, including technology-related interactions, could trigger or perpetuate trauma among end users. To mitigate the risks, both technology developers (particularly designers) and health providers should consider design choices and implementation strategies that not only prevent trauma but also promote users’ emotional well-being.

## Introduction

Digital health technologies are increasingly used as complementary or alternative means of seeking potentially sensitive and stigmatized sexual and reproductive health services [1,2]. While there is no universally accepted definition of digital health, it is generally conceptualized as the use of information and communications technologies in medicine and other health professions to manage illnesses, improve health, and health care delivery [3]. The increasing adoption of digital health is revolutionizing how sensitive and stigmatized sexual and reproductive health services are accessed and delivered [2]. Digital health technologies are enabling individuals to access confidential, evidence-based information and services such as contraception management, fertility tracking, sexually transmitted infection (STI) prevention, and telemedicine consultations for abortion services [4-6]. These tools (such as telemedicine and other virtual care modalities like video appointments) have expanded access to care for marginalized populations (ie, those who regularly experience disproportionate burden of illness due to structural disadvantage, stigma, and discrimination), including those in remote areas or with limited access to traditional health care facilities. Health care systems are increasingly integrating digital health platforms to improve patient engagement, provide personalized care, and address disparities in sexual and reproductive health outcomes. This adoption is driven by advancements in technology and a growing recognition of the importance of user-centered, accessible solutions.

Despite the benefits of digital health technologies for sexual and reproductive health services, there are increasing concerns that such platforms could facilitate some form of harm that can lead to emotional trauma among end users [7,8]. Trauma resulting from the use of digital health technologies (herein referred to as technology-related trauma) can occur depending on how technologies are designed, deployed, or used [7]. While there is not yet a universally accepted definition of technology-related trauma, it is generally conceptualized as technology-enabled activities, interface appearances, or features that leave a user in emotional distress following the use of digital technology [7]. Technology-related trauma can be orchestrated intentionally through negative online activities or inadvertently through interface features that remind people of prior traumatic circumstances [9,10]. For instance, interactive technologies that expose people to online scams, sextortion (sharing of nude pictures on sexual health–related webpages), identity theft (using someone’s personal information for soliciting or selling sex products), and cyberbullying (insulting and condescending language in online forums) could result in short-term or long-term emotional trauma, and sometimes, suicidal thoughts for people [11-14].

Digital technologies can also trigger emotional trauma through interface features that may be used to harass, coerce, or stalk someone [15]. These sorts of abuses can range from intimate image abuse and the use of geolocation features for stalking to the use of Internet of Things devices to manipulate people into doubting their sanity [16]. For instance, perpetrators of intimate partner violence can exploit digital health technologies by installing spyware on their partners’ devices, compromising their accounts and the potential safety of their victims [15]. Also, downloading health applications that collect, store, or transmit users’ location and other personal identifying information can cause short and long-term emotional distress that could result in traumatic outcomes [17]. For people who survived trauma, viewing certain sexually explicit content that they consider to be inappropriate can retraumatize them and further create technology-related cycles of retraumatization each time the content is viewed [18]. Even seemingly neutral user interface design elements developed in line with established design guidelines (eg, the use of different colors and images) can trigger emotional trauma in people with negative prior experiences or a negative attitude towards the said elements [19]. Bonell et al [20] termed these sorts of occurrences the “dark logic” of public health interventions, while Ziebland et al [21] also described it as the “paradoxical consequences of technologies,” where interventions designed for treating a particular condition inadvertently deliver a contrary outcome.

While technology-related trauma can occur in any technology-based intervention, its occurrence may be more common in technology interventions that are used for accessing sexual and reproductive health services that are perceived as embarrassing, stigmatizing, and difficult to discuss face-to-face [7,22]. People who seek highly stigmatized sexual and reproductive health services like STI testing, contraception, and abortion services may be prone to technology-related trauma because such services are already associated with an immense amount of societal stigma and shame [7,23]. Due to the stigma associated with visiting conventional sexual and reproductive health facilities, digital health is often used as a complementary or alternative intervention for people who want to access sexual and reproductive health services.

Given the stigma and shame already associated with such services, any negative event experienced via technology-based interventions could either trigger or perpetuate such prior trauma. For instance, interventions designed to increase adherence to HIV antiretroviral drugs or interventions for promoting contraceptive use can trigger traumatic experiences when people’s personal information collected through these interventions is deliberately or inadvertently disclosed. Furthermore, the use of abortion-related technologies can generate hate and antichoice activities that may lead to traumatic feelings [22,24]. Other digital health such as menstrual tracking apps, web-based virtual consultation services, and mobile apps has been shown to incorporate surveillance and social control mechanisms to depict sexual and reproductive health services like abortion in a negative light [25]. Given the stigma associated with sexual and reproductive health services and the concurrent increase in digital technology adoption [26], any additional trauma resulting from the use of technology interventions could create and exacerbate health inequities, particularly for marginalized populations who rely on technology-based interventions as complementary or alternative means of seeking health care. Despite the threats of technology-related trauma in sexual and reproductive health technologies, researchers have yet to explore how trauma manifests in sexual and reproductive health-related technologies. This study sought to address this gap by generating an explanatory model of how digital health technologies can contribute to or perpetuate trauma among end users.

## Methods

### Study Design

We adopted the Charmaz constructivist grounded theory approach [27] to generate an explanatory model of how emotional trauma manifests in the contexts of sexual and reproductive health technologies. Grounded theory was considered an appropriate approach to exploring this phenomenon as little is known about how emotional trauma can be orchestrated through sexual and reproductive health technologies.

### Ethical Considerations

Ethical approval was obtained from the University of British Columbia Behavioral Research Ethics Board (H23-02934). Written, fully informed consent was obtained from all participants in adherence to the requirements outlined in the Declaration of Helsinki. The interviews were deidentified before analysis. At the end of each interview, participants were provided with CAD 30 (approximately US $23) in the form of an Amazon gift card as honoraria for their time.

### Study Population, Eligibility, and Recruitment Approaches

The definition of trauma in this study is specific to emotional and psychological trauma, which is different from physical trauma (ie, injuries or body wounds produced by sudden physical injury from impact, violence, or accident). Psychological trauma is used to describe the emotional reactions that occur in response to extraordinary frightening or distressing events [28]. The population for this study comprised people who use digital health technologies like web-based platforms and mobile apps to access sexual and reproductive health services or information across provincial cities in western Canada. Digital health technologies are used to refer to government and not-for-profit regulated web-based platforms or mobile phone apps that are used to provide sexual and reproductive health services including contraception, abortion, STI testing, medical appointments, and teleconsultation services. Participants were recruited from 2 main sources: ReachBC and Options for Sexual Health Clinics. ReachBC is a provincial web-based platform that connects British Columbia volunteers and researchers to facilitate health research in the province of British Columbia. Options for Sexual Health is the largest nonprofit sexual health organization in Canada and has more than 50 clinics in British Columbia. For this study, the recruitment notice was posted on ReachBC and shared across 7 Options for Sexual Health participating clinics. Individuals who expressed interest contacted the research team, and their eligibility was confirmed prior to scheduling an interview.

To be eligible for inclusion, participants must: (1) have used a web-based platform or an mHealth tool to access any of the sexual and reproductive health information or services outlined above within the last year, (2) be aged ≥19 years, and (3) be able to speak English.

### Data Collection

Data were collected through individual semistructured interviews conducted via Zoom (Zoom Communications, Inc). All interviews were conducted by the first author. Before delving into the interview questions, we asked participants to complete a brief verbal survey about their demographic characteristics. The interview questions addressed topics related to how digital health technologies could cause emotional trauma among end users. Specifically, participants were asked questions related to their experiences in using digital health technologies to access sexual and reproductive health services, the challenges they encountered when using digital health technologies, and how the use of such technologies could cause or perpetuate trauma. They were also asked questions about how the features and interface appearance of digital health technologies could perpetuate historical and existing vulnerabilities among people with sexual and reproductive health conditions or disorders. Data collection occurred until we reached saturation by informational redundancy (when new data repeats what was previously said).

While we did not seek to actively recruit people with prior trauma, there was a tendency for some people with prior trauma to choose to participate in the study. Given this understanding and the fact that some participants might have had prior trauma related to sensitive health topics like contraception, abortion, and STI testing, we embedded trauma-informed practices throughout the research processes: before, during, and after the data collection [29,30]. Before data collection, the first and second authors were trained in trauma-informed care practices on how to communicate with and identify signs of distress among participants. We also spelled out the potential question areas to address during the interviews as well as the interview procedures on the consent form. Two community partners and an expert in trauma-informed care reviewed the interview guide and provided feedback before the data collection began. We also co-created and distributed a self-care resource to each participant 3 days ahead of the interview. The participants were asked to review the self-care resources before and after the interviews. In these resources, we provided contacts of counselors to reach out to in case of any distress. Recognizing that some participants might be living with perpetrators of abuse, we encouraged them to choose locations that are safe and convenient to step out when the need arises. *During the data collection,* we asked participants to take a break or end the interviews if they felt uncomfortable. We periodically checked in with the participants to see if they were doing well or showed any signs of discomfort or tiredness on their part. To ensure anonymity and promote participants’ freedom of expression, participants were given the option to turn off their videos if desired. However, the interviewer had their Zoom turned on throughout the course of the interviews. After the data collection, we followed up with each participant in 2 days and 1 week to see how they were doing. We also provided them with contacts of mental health services within their catchment areas to reach out to in case they develop any signs of emotional distress following the interview. All the interviews lasted between 45 and 60 minutes. Data collection and analysis occurred concurrently. As recruitment and data analysis proceeded, we used the emerging concepts from the preliminary findings as a guide to recruiting additional participants to fill uncertainties in the data and build on the emerging theoretical model. These additional participants reflected people with diverse lived experiences as well as diversity in age, sex, and gender identity. The interviews were audio-recorded, transcribed using the web-based transcription services (Temi, Inc), deidentified, and then exported into NVivo (Lumivero) for analysis.

### Data Analysis

The first and second authors reviewed the audio transcripts immediately after each interview. The audios were then transcribed, and each textual transcript was compared with its corresponding audio file. A trauma-informed lens also informed ethical handling of the data, prioritizing participant confidentiality while minimizing harm. Consistent with the constructivist grounded theory approach, we generated inductive codes through initial coding, focused coding, and theoretical coding [27]. In initial coding, the first and second authors “broke down the raw data” into parts by labeling each data segment with codes. As the data analysis progressed, we compared the data to identify any similarities or differences among participants of different ages, sexes, and gender identities. The data analysis occurred concurrently with data collection, and additional data were used to modify the codes. In focused coding, we sorted, integrated, and organized the most significant and frequent initial codes into the core categories. We sought explanations for differences, explored associations between the categories, and further examined which codes contribute most to the emerging categories. In theoretical coding, we refined the categories and related them to one another to construct a theoretical understanding of the processes by which digital technologies could foment or perpetuate trauma among end users (Textbox 1). Our understanding of trauma informed our data analysis by fostering sensitivity to participants’ experiences of digital technologies and how that may shape their narratives. We contextualized participants’ responses within broader systemic and relational dynamics, recognizing the impact of prior trauma and power imbalances between the participants and the researchers. Recognizing that participants might choose to filter or withhold information due to fear, mistrust, or discomfort rooted in past trauma, we paid attention to areas in the transcripts where participants may be silent, avoiding some questions or withholding some information. Analyzing the data from a trauma perspective helped us to fill in uncertainties and confirmed participants’ narratives regarding some topic areas that could be regarded as sensitive.

Textbox 1. **An overview of the main categories and coding process.**

**Thematic area: inappropriate content display**

- Traumatic content
  - Depicting explicit images on digital platforms
  - Exposure of personal or nude photos
  - Sudden exposure to sexualized content (images and videos)
  - Not warning people about sexually explicit content
- Misrepresenting identity
  - Gender misrepresentation in online platforms
  - Wrong or inappropriate translation of content in platforms
- Explicit content
  - Profane advertisement via online platforms
  - Profane images

**Thematic area: data and security breaches**

- Cyber-attack concerns
  - Concerns about cyber-attacks and lawsuits
  - Hacking and phishing attempts
  - Concerns about possible data breaches
  - Concerns with creating profiles on online platforms
- Identity exposure
  - Concerns with exposing personal information to health providers
- Confidentiality concerns
  - Lack of privacy and anonymity of digital resources
  - Lack of privacy when accessing via public computers
  - Revealing confidential information

**Thematic area: navigational challenges**

- Dense interfaces
  - Inability to find the desired information
  - Difficulty finding information in a stressful time
  - Crowded spaces in digital platforms
  - Difficulty in understanding content
- Technical difficulties
  - Technophobia in using technology
  - Going through multiple menus to locate information

**Thematic area: technology-related targeted campaigns**

- Identity theft
  - Linking sensitive issues to peoples’ names
  - Using personal information for fraud
  - Using people’s data against them
- Tracking concerns
  - Concerns about cookies
  - Soliciting for sensitive information on web platforms
- Misinformation and disinformation
  - Tracking personal information with targeted advertisement
  - Fake and misleading information on sexual minorities
  - Negative stereotyping
  - Anti-lesbian, gay, bisexual, trans, intersex, or queer (LGBTIQ) activities on web platforms

**Thematic area: technology-related depersonalization of sensitive sexual health interactions**

- Trauma-triggering communication
  - Reminding people of trauma triggers
  - Unsympathetic communication with care providers
  - Victim blaming during online communications
- Cultural insensitivity
  - Lack of language and cultural sensitivity
  - Nonpersonalized communication and information
  - Not understanding people’s prior trauma history
  - Trivializing sexual health (sexually transmitted infections and abortion)
  - Normalizing people’s suffering through technology

## Results

### Demographic Characteristics

A total of 25 participants took part in the study, of which 16 (64%) of the participants identified as women, 5 (20%) identified as men, and 4 (16%) identified as nonbinary. The participants’ ages ranged from 23 to 67 years, with a mean age of 35 (SD 12) years. All participants indicated having used digital technologies to access sexual and reproductive health services, including STIs, contraception, and abortion services, within the last year. Even though participants were not asked about prior trauma experiences, approximately 68% (17/25) directly or indirectly revealed that they have experienced a prior event in the past that they consider to be traumatizing. While the other 32% (8/25) did not indicate prior trauma, we could not tell whether they had prior trauma, as some participants might choose not to disclose their trauma history.

### How Trauma Manifests in Digital Health Platforms

**Figure 1.**
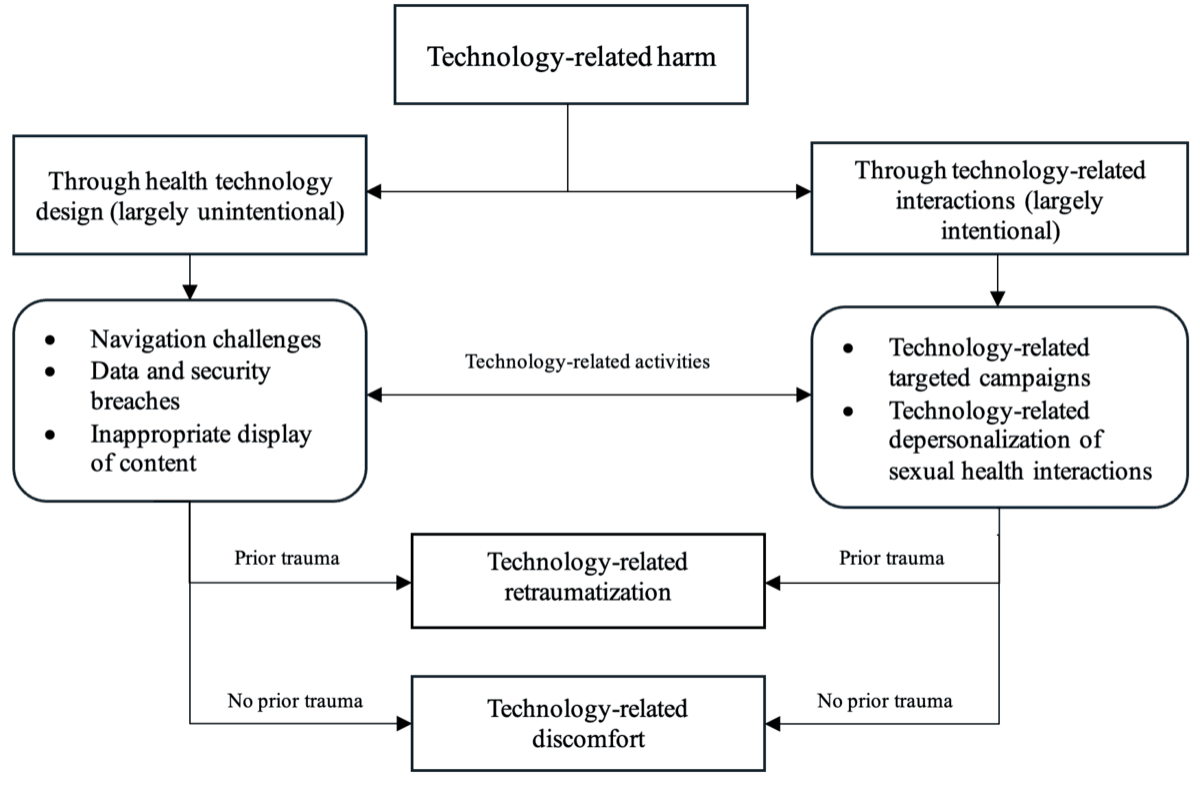
An explanatory model of technology-related trauma in sexual and reproductive health.

In this study, we constructed a theoretical model illustrating how digital health technologies can facilitate trauma among end users. Our model shows that digital health platforms can harm patients seeking sexual and reproductive health services or information in 2 primary ways: digital platform design features (ie, navigation challenges, data and security breaches, and inappropriate display of content) and technology-related interpersonal interactions (targeted campaigns and depersonalized digital health interactions). However, whether these technology-related harms result in trauma, however, depends on an individual’s prior experiences of trauma related to sexual and reproductive health services. People with prior trauma related to sexual and reproductive health services who happen to experience these negative activities are likely to experience retraumatization, while people with no prior trauma are likely to experience technology-related discomfort. Figure 1 presents the theoretical model of sexual and reproductive health technology-related trauma developed in this study. The next sections elaborate on how each activity manifests as technology-related trauma.

### Through Health Technology Design (Largely Unintentional)

#### Overview

This section presents how certain user interface design features may contribute to psychological or emotional trauma among individuals accessing sexual and reproductive health services through digital platforms. These activities were considered largely unintentional and could occur during the design and development stages of digital health technologies. These activities are considered largely unintentional, as they typically arise during the design and development stages when technology developers act with good intentions but may not fully recognize the potential for retraumatization of users.

#### Inappropriate Display of Content and Lack of Representation in Digital Platforms

The participants indicated ways in which the presentation of content on digital health platforms could retrigger emotional trauma or cause emotional discomfort among people with sexual and reproductive health conditions or disorders. In particular, imagery, videos, and language perceived as vulgar were described as potentially retraumatizing when they evoked memories of past traumatic experiences. Some participants recounted how they got distressed when such imagery, videos, or language were suddenly exposed to them without any prior warning. One participant who identified as a woman indicated how awkward and distressful she felt upon being suddenly exposed to a picture of female genital mutilation (ie, a complete or partial removal of the female external genitalia). Participants noted that certain technology-based interventions, such as those designed to support individuals with STIs or to assist survivors of intimate partner violence or sexual assault who become pregnant, could inadvertently retraumatize users by triggering reminders of their past experiences. One participant reiterated how sexual assault victims could be retraumatized via digital technologies by stating that:

if somebody who got pregnant as a result of sexual assault were to go to a website and look for help or look for sexual health services. And all of a sudden, they started seeing pictures, videos, or stories of sexual assault. Those things could all potentially be triggering because they are reminding them of what happened to them.

While the display of explicit content could retraumatize people by reminding them of their prior experiences, participants varied in how they perceived and responded to this possibility. Sudden exposure to explicit content was reported to trigger emotional trauma among participants with prior real-life experiences, whereas for those without such histories, these images were more likely to cause emotional discomfort rather than trauma.

In addition to the inappropriate display of content on digital platforms, some participants, particularly 2-spirit, lesbian, gay, bisexual, transgender, queer, intersex, and gender-diverse individuals, felt that they are often not adequately represented and, in some situations, misrepresented on digital health platforms. They indicated that such misrepresentation could perpetuate existing harmful practices that they already face in society and could trigger emotional trauma when they visit digital health platforms. The participants also indicated that they could feel some emotional discomfort and, in some cases, distress when well-intentioned educational messages on digital health platforms conflict with their situation. One participant likened it to seeing messages about baby care or the need to have children after having had an abortion. They revealed how they felt uncomfortable after seeing an advert by stating that:

I opened this website and I saw something, and it was like life begins at conception. For someone who had an abortion, I was just so disgusted…. and that goes to perpetuate whatever traumatic experiences I might have had during the abortion because I am thinking, like maybe, I should have kept this kid even though I can’t financially and emotionally support this kid. It’s just like thinking back and all that trauma it comes with.

#### Privacy, Data, and Security Breaches in Digital Platforms

Participants also described how unsecured digital health design features could expose personal information stored on digital platforms to unauthorized access and potential misuse. Participants noted that data breaches in digital health can cause profound emotional trauma for patients with sexual and reproductive health disorders due to the highly sensitive nature of the information involved. They indicated that the unauthorized disclosure of sensitive information, such as infertility, sexually transmitted infections, or abortion history, can result in stigma, shame, and fear of judgment from peers, employers, or family members. The breach of privacy may also exacerbate preexisting mental health challenges, including anxiety and depression, as patients worry about how their data could be misused. For people with prior trauma, the mere thought of data and security breaches, phishing attempts, and possible cyber-attacks was considered distressful and could trigger past traumatic events. One participant indicated how data breach concerns could retraumatize them by stating that:

My concern just comes down to privacy, how much information is revealed, what happens with the data that we enter into these systems, where and how is that stored? And you know, those are the kinds of things that I want to know because it worries me that it could get into the wrong hands. And that includes an unfriendly government. And when you add all these layers on top of those really sensitive things about yourself, it could be scary and traumatic. I mean, let’s not mess around here.

The participants indicated that the loss of confidentiality in digital platforms can erode trust in health care systems and may discourage individuals from seeking the necessary care, ultimately worsening their emotional and physical health challenges. While participants noted that data breaches could retraumatize those with prior trauma, they were more often perceived as causing emotional distress among individuals without a reported trauma history. Participants, particularly those who use public computers, expressed concern that their personal information could be exposed and their identities revealed when accessing services in such settings. Concerns about information exposure were especially pronounced among individuals seeking sexual or reproductive health information.

#### Navigation Challenges in Digital Platforms

The findings also show that design-related issues, such as navigational challenges on digital platforms, can trigger or exacerbate existing trauma for some individuals seeking sexual and reproductive health services, particularly those considered private, stigmatizing, or uncomfortable to discuss in face-to-face settings. The participants noted that dense, cluttered user interfaces and excessive navigation steps could create delays and heighten anxiety for individuals who are already traumatized. For people with prior trauma seeking sensitive sexual and reproductive services, encountering poorly designed interfaces, unclear instructions, or inaccessible resources can exacerbate emotional distress. Participants indicated that their inability to quickly find confidential, reliable information or services may heighten feelings of isolation and stigmatization, making them question their decision to seek help online. One participant cited how the presence of many icons and the detailed registration processes on a web-based platform could add to their anxiety by stating that:

Emotionally, I think it just adds to your stress already. You could already be quite worked up and upset or maybe if it’s an emergent situation like I needed a pregnancy test or I’m looking to have an abortion. So, there’s just a lot of emotions involved. And then to kind of add this added stress of how to figure it out on your own and then have follow many steps and to call this or go here and book it.

According to the participants, this “compounded stress” can lead to avoidance behaviors, where individuals delay or forgo critical health care services altogether, further impacting their mental and physical well-being.

### Through Technology-Related Interactions

#### Overview

Our model further illustrates that technology-related retraumatization can occur through interactive activities, specifically (1) targeted digital campaigns and (2) the depersonalization of sensitive sexual health interactions. This thematic category was classified as largely intentional, as such activities often occur during patients’ use of digital health technologies and may be deliberately orchestrated to cause harm.

#### Technology-Related Targeted Campaigns

Participants expressed concern that technology-related targeted campaigns could trigger or perpetuate trauma among end users. These concerns applied to campaigns delivered through both regulated sexual health digital platforms and social media. Some participants reported feeling distressed when pornographic images unexpectedly appeared on sexual health-related digital platforms or were targeted at them via social media. The participants also indicated that people who sought stigmatized services like abortion could get traumatized if baby products or pregnancy-care products suddenly pop up. One participant stated:

On the internet these days, everything you do is being tracked, then repackaged, and then used to try to sell you things. For someone who just lost their baby or had an abortion being sent an advert like baby products, how would you want them to feel after they have gone through this ordeal? And there are certain things, especially with trans people where if you have to put your legal name down in these things, you are dead naming yourself, so you’re having to use the name that causes you harm.

Participants also indicated that targeted advertisements or content based on sensitive browsing histories or health-related searches could make them feel threatened or exposed, as if their private struggles were being exploited for profit. This was particularly distressing for individuals dealing with stigmatized issues such as sexually transmitted infections, infertility, abortion, or contraception. The participants indicated that constant exposure to unsolicited advertisements can serve as a reminder of personal challenges, triggering anxiety, embarrassment, or shame. Furthermore, the fear of being “found out” or judged if someone else sees such targeted advertisements adds another layer of psychological distress, eroding trust in digital platforms and health care systems. Participants also noted that digital health technologies can be used to target minority groups with misinformation and negative stereotypes. They highlighted that sexual minority individuals seeking services such as abortion are particularly vulnerable to such technology-related misinformation, given societal disapproval of such services.

### Technology-Related Depersonalization of Sensitive Sexual Health Interactions

Participants also expressed concerns about how interactive digital health platforms could depersonalize the care process and lead to patient-provider practices that could inadvertently retraumatize people. The participants noted that health care professionals who may not understand the social and emotional history of their patients might communicate as though “everything is normal,” while some providers are likely to ask questions that remind people of their prior trauma or seek to blame them for their predicaments. One participant indicated that:

People could get retraumatized if healthcare professionals communicate in ways that seek to blame or remind them of their past experiences, like how they got infected with STIs, how they got pregnant, or why they are seeking contraception or abortion services.

Participants noted that digital health technologies, particularly virtual care such as telemedicine, often limit providers’ ability to gain a detailed understanding of their patients. Without the ability to see facial expressions in some technology-related interactions, health professionals may not be able to convey the needed empathy for a patient who might have survived trauma. Such people could be retraumatized if virtual communications do not convey the needed empathy. One participant stated:

Sometimes when you are interacting with health providers online, you don't think you're fully heard. If it's just a chat room or a phone call, the healthcare professional might not understand or know what state of mind this person is in because they can't see you. Let's say if they're in a really dire crisis mode and the healthcare professional didn't really know it because they're just asking a question. They might trigger something within them.

In addition to the possibility for retraumatization via technology-related depersonalizations, the participants were also concerned that technology-related peer-to-peer interaction channels like chatrooms and messaging boards could trigger and perpetuate trauma when people infiltrate these platforms with the intent of harming other participants.

## Discussions

### Principal Findings

Digital health technologies are increasingly recognized as offering opportunities for advancing sexual and reproductive health and services [2,6]. At the same time, these technologies can also serve as conduits through which trauma can be triggered, perpetuated, and exacerbated [21,22]. While various trauma-informed approaches have been proposed in the technology design arena [31-34], they are often not grounded in a foundational understanding of how technologies can cause trauma, nor tailored to the distinctive elements of sexual and reproductive health. To our knowledge, this study is the first to develop a theoretical model illustrating how emotional trauma can manifest within sexual and reproductive health technologies. Our model suggests that technology-related harm may largely arise either unintentionally through health technology design or intentionally through technology-mediated interactions. However, whether these technology-related activities result in emotional trauma depends on whether people have prior trauma related to sexual and reproductive health. For people with prior trauma, exposure to inappropriate content, data and privacy breaches, targeted campaigns, navigational challenges, or nonpersonalized interactions can result in retraumatization when these experiences mirror or reinforce their past trauma. Conversely, individuals without such histories may only experience emotional discomfort from these technology-related encounters, though the impact is less likely to be retraumatizing. The differences in opinions on how technology-related harms can lead to trauma in digital health platforms reflect the assertion that not everyone who is exposed to a traumatic event will eventually be traumatized [35]. That is, technology experiences can elicit harm, but such harm may not necessarily translate into trauma for some group of people.

Our study also confirmed the assertion that design elements that might be considered minor by the design team or some conversations that are considered usual in patient-provider interactions could still end up retraumatizing some people [36]. Other design elements developed with the best of intentions could still end up retraumatizing people if they find such content undesirable or reminding them of their prior trauma [19]. For instance, some abortion-related educational content displayed on digital health platforms may remind people of abortion trauma [37]. Although such design features may be viewed as standard by intervention developers, some are likely to trigger or perpetuate psychological and emotional trauma among equity-deserving populations who have experienced personal, relational, or intergenerational trauma, including indigenous peoples, individuals experiencing homelessness, those with disabilities, or survivors of intimate partner violence [7,38-40]. For such people, technology-related trauma may occur on top of other traumas, thus potentially exacerbating their harm.

One important finding from this study was how digital health–related interactions can intentionally or inadvertently trigger or perpetuate technology-related trauma. While previous studies revealed how digital health technologies can be deliberately used to cause trauma [14], our findings highlight the surprising reality that routine digital health interactions can also inadvertently retraumatize individuals. The potential for such retraumatization may stem from the limited opportunities in virtual care for health care professionals to fully recognize or understand a patient’s trauma history. This lack of recognition of patients’ traumatic past can lead to impulsive remarks that can be deeply hurtful, or at best, unhelpful to the patient’s situation [41]. Even when empathy is conveyed, the lack of body language in digital communications might make it easier for messages to be interpreted as unsympathetic or harsh [42]. Furthermore, digital communication may lack the nuance needed for a sensitive topic like sexual health—making some information appear abrupt or dismissive to patients. This assertion is supported by prior studies that noted that the absence of verbal and nonverbal cues, body language, and tone in virtual care technologies can hinder the development of trust and rapport between health care providers and their patients [43,44]. For people who survived trauma, the inability to fully capture patients’ verbal and nonverbal cues may not only make it challenging for providers to recognize and respond to signs of distress but may also leave patients feeling unsupported or make them struggle to express their emotions or disclose their trauma history [45]. For people with prior trauma, this could go a long way to hinder communication, affect rapport building, and make the health-seeking process incredibly difficult and time-consuming for the patient, hindering the trauma-healing process [46].

In addition to trauma arising from digital health–related interactions, concerns about data and privacy breaches, misinformation, and unexpected exposure to explicit digital content were also reported to retraumatize individuals. These activities can erode trust and make technology users feel skeptical about the authenticity of health advice and product recommendations, while causing them to question the motives behind the information they receive. Such targeted campaigns can make people feel as though they are constantly being watched and analyzed, leading to discomfort and distress over the potential loss of their anonymity and privacy [47,48]. The concern that one’s information is under surveillance could also make them develop feelings of paranoia and fear. Constant worry about the misuse of personal information can lead to chronic anxiety or depressive episodes, potentially leading to mistrust and nonadoption of digital health technologies. In addition, cultural insensitivity in virtual interaction with patients, particularly those from minority groups, might inadvertently perpetuate trauma. For instance, when virtual interactions fail to recognize or respect cultural values, norms, and experiences, patients may feel misunderstood, marginalized, or dismissed, potentially exacerbating existing trauma and reducing trust in digital health services.

The findings indicate that digital health interventions may represent an emerging and underrecognized source of trauma, particularly affecting individuals who experience stigma, discrimination, or inequitable access to health care services. Therefore, health care systems that develop digital health interventions should consider how digital health design features and digital health–related interactions could trigger or perpetuate emotional trauma. Given the growing use of sexual and reproductive health digital health technologies among survivors of trauma [33], there is an urgent need to pay attention to interface features that could end up retraumatizing the very people they intend to help. Special attention should be given to user-centered design practices by fully engaging with people with trauma during the design of digital health technologies. Digital health intervention developers within the health care system should also consider integrating privacy and security measures, including content warnings and user control on digital platforms, paying attention to inclusive representation and cultural sensitivity, integrating accessible design, a continuous evaluation process, and integration with in-person services are critical to mitigating technology-related trauma [24]. Textbox 2 shows a summary of considerations for trauma-informed digital technologies in sexual and reproductive health.

Textbox 2. **Key recommendations for trauma-informed digital health technologies.**

**Design considerations**

- Privacy and security measures integration
- Inclusive representation and cultural sensitivity
- Clear navigation pathways
- Trauma-informed language and imagery guidelines
- Implementation of content warnings and user controls

**Implementation considerations**

- Provider training in trauma-informed digital communication
- Integration with in-person services
- Regular user feedback mechanisms
- Cultural safety evaluation
- Privacy impact assessments

**User-support considerations**

- Clear opt-out pathways
- Accessible help resources
- Alternative service options
- User control over data-sharing
- Support for diverse communication preferences

### Limitations

This study was based on a small sample of 25 participants that was limited to one geographic region in western Canada. These limitations highlight the need for future research to engage participants from more diverse cultural backgrounds and from regions across Canada and beyond to ensure that the emerging theory on technology-related trauma reflects cross-cultural and geographical nuances. While some of our participants reported (directly or indirectly) some traumatic experiences in the past, we could not ascertain that their views represent varying forms of traumatic experiences that tend to affect people who access sexual and reproductive health services. Despite these limitations, the findings offer important and timely implications for designing and using digital health technologies that transcend beyond the study settings.

### Conclusions

Technology-related trauma in sexual and reproductive health digital platforms underscores a critical intersection of healthcare innovation and patient vulnerability. While digital platforms offer unprecedented opportunities for education, support, and access to services, they also pose significant risks of retraumatization and emotional harm. Privacy concerns, the potential for exposure to triggering content, and the depersonalization of sensitive sexual health interactions can retraumatize individuals already navigating complex and sensitive sexual and reproductive health challenges. Given this foundation knowledge of the emerging threats of digital health technologies, health care providers, technologists, and policy makers need to work collaboratively to mitigate these risks, for example, by implementing robust security measures, promoting digital literacy, and ensuring that the human element of care is prioritized in digital health practices. By doing so, the potential harms of technology-related trauma can be minimized, allowing people to realize the full potential of sexual and reproductive health–related digital platforms without compromising patients’ emotional safety. We anticipate that the findings of this study will produce a new understanding that will help inform recommendations for mitigating technology-related trauma in digital health.

## Abbreviations

STI: sexually transmitted infection
